# Multi-omics and machine learning for the prevention and management of female reproductive health

**DOI:** 10.3389/fendo.2023.1081667

**Published:** 2023-02-23

**Authors:** Simmi Kharb, Anagha Joshi

**Affiliations:** ^1^Department of Biochemistry, Postgraduate Institute of Medical Sciences, Rohtak, Haryana, India; ^2^Computational Biology Unit (CBU), Department of Clinical Science, University of Bergen, Bergen, Norway

**Keywords:** pregnancy, endocrinology, metabolic syndrome, pregnancy complications, omics technologies, e-health, biomarkers

## Abstract

Females typically carry most of the burden of reproduction in mammals. In humans, this burden is exacerbated further, as the evolutionary advantage of a large and complex human brain came at a great cost of women’s reproductive health. Pregnancy thus became a highly demanding phase in a woman’s life cycle both physically and emotionally and therefore needs monitoring to assure an optimal outcome. Moreover, an increasing societal trend towards reproductive complications partly due to the increasing maternal age and global obesity pandemic demands closer monitoring of female reproductive health. This review first provides an overview of female reproductive biology and further explores utilization of large-scale data analysis and -omics techniques (genomics, transcriptomics, proteomics, and metabolomics) towards diagnosis, prognosis, and management of female reproductive disorders. In addition, we explore machine learning approaches for predictive models towards prevention and management. Furthermore, mobile apps and wearable devices provide a promise of continuous monitoring of health. These complementary technologies can be combined towards monitoring female (fertility-related) health and detection of any early complications to provide intervention solutions. In summary, technological advances (e.g., omics and wearables) have shown a promise towards diagnosis, prognosis, and management of female reproductive disorders. Systematic integration of these technologies is needed urgently in female reproductive healthcare to be further implemented in the national healthcare systems for societal benefit.

## 1 Endocrinology through the female life cycle

Female reproductive life starts from fetal life, spanning childhood and puberty, to the reproductive years with final ovarian follicle depletion and menopause. The ovaries maintain the health of the female reproductive system, and they secrete two main hormones—estrogen and progesterone. Reproductive longevity is essential for fertility and influences healthy aging in women. Energy homeostasis and gonadal steroid levels control the female reproductive life cycle. Genetic and environmental factors can cause perturbations to it, with resultant increased body fat and insulin resistance, leading to metabolic syndrome. Diseases associated with ovaries include ovarian cysts, ovarian cancer, certain menstrual cycle disorders, and polycystic ovary syndrome (PCOS). Similar to every organ system, the female reproductive system is dependent on energy homeostasis. Premature adrenarche, PCOS, and gestational diabetes can occur due to shifting to androgenic gonadal steroid levels and reduced insulin sensitivity. Changes or abnormalities in ovarian function are related to environmental, genetic, and epigenetic factors, and they coexist with metabolic abnormalities.

## 2 Female reproductive and pregnancy-related complications

The two most common reproductive disorders in women are PCOS and endometriosis. PCOS is a disorder characterized by hyperandrogenism, ovulatory dysfunction, and polycystic ovaries. Hyperandrogenism is recognized as a key diagnostic factor, in combination with other symptoms of the syndrome (1). There are some disparities in diagnostic approaches worldwide. According to the NIH (National Institutes of Health), PCOS is diagnosed by the presence of both hyperandrogenism and olgio/amenorrhoea (2). The incidence of PCOS varies according to the diagnostic criteria. Women with hyperandrogenic chronic anovulation (i.e., NIH criteria) make up approximately 7% of reproductive-aged women. The Rotterdam criteria require the ultrasonic appearance of the polycystic ovary for PCOS diagnostics (3) and the presence of one polycystic ovary is sufficient to provide the diagnosis (4). It increases the prevalence of PCOS in women with normogonadotropic anovulation to 91% from 55% using the NIH criteria (5). All diagnostic approaches recommend that secondary causes (such as adult-onset congenital adrenal hyperplasia, hyperprolactinemia, and androgen-secreting neoplasms) should first be excluded. Machine learning approaches show promise towards PCOS detection. A machine learning technique applied on ovary ultrasonography scans could reliably predict PCOS using image data (6). Despite the fact that both PCOS and endometriosis impair female reproduction and can lead to infertility, they are very different. Endometriosis is a gynecologic disorder characterized by the presence of endometrial tissue outside the uterine cavity, and PCOS is caused by a hormonal imbalance. It has been hypothesized that endometriosis and PCOS represent extreme (and diametric opposite) outcomes of variation in HPG axis development and activity, with endometriosis mediated mostly by low prenatal and postnatal testosterone, while PCOS is mediated by high prenatal testosterone (7). Endometriosis affects approximately 5% of women in reproductive age and often presents with pelvic pain, infertility, or an ovarian cystic mass in these women. Growth of endometrial lesions is stimulated by estrogen and inhibited by androgens. Genomics analysis revealed the role of aromatase, which activates the promoter of steroidogenic factor-1 and subsequently increased the local conversion of androgen to estrogen (8).

Pregnancy is a highly resource-intensive stage in the female reproductive life cycle. Most women suffer from physical and/or psychological disturbances during pregnancy. Therefore, pregnancy without any complications is in fact not so common. We further elaborate on some of the common pregnancy complications.

### 2.1 Preeclampsia

Preeclampsia complicates 2%–8% of pregnancies globally and is responsible for over 20% of maternal deaths. Preeclampsia is a multi-system disorder of pregnancy and is a leading cause of both maternal morbidity and neonatal mortality (9, 10). Risk factors include extremes of maternal age, twin paternity, nulliparity, increased BMI, increased systolic and diastolic blood pressure during early pregnancy, and the presence of gestational diabetes. Preeclampsia is the main maternal risk factor for low-birth-weight newborns and/or intrauterine growth restriction and/or fetal death (11). Currently, there is no single reliable, cost-effective screening test for preeclampsia. Management of preeclampsia depends on gestational age and its severity and its basic objectives are supportive (including termination of pregnancy or uneventful delivery and birth of infant) since the conditions return to normal after the delivery of the baby. ACOG guidelines for the management of preeclampsia include monitoring and management of blood pressure, and risks of expectant management in the presence of severe features, namely, pulmonary edema, MI, stroke, ARDS, coagulopathy, renal failure, and retinal injury. Medications to treat severe preeclampsia include antihypertensive drugs, anticonvulsant medications (magnesium sulfate), and corticosteroids. Preeclampsia is multifactorial in origin, and genetic, immunologic, and environmental risk factors influence the development of preeclampsia. The etiology and pathogenesis of preeclampsia are not yet fully understood and numerous theories on the pathophysiological mechanisms are available (12). The dominating theory of the origin of preeclampsia is defective placentation and insufficient penetration of trophoblasts, which result in impaired maternal blood flow through narrow spiral arteries (13) and the reason for this defective trophoblast behavior is not known. It could be due to immunologic dysfunction causing unwanted hampering of normal trophoblast activity, and other mechanisms could be placental and endothelial dysfunction, immunological maladaptation to paternal antigens, and exaggerated systemic inflammatory response (14–16).

### 2.2 Gestational diabetes

Gestational diabetes mellitus is defined as carbohydrate intolerance resulting in hyperglycemia, including impaired glucose tolerance with first onset or detection during pregnancy (17). The prevalence of gestational diabetes is thought to be between 2% and 25% worldwide. Moreover, the prevalence of GDM is on the rise likely due to increased maternal age and BMI during pregnancy. Risk factors of GDM include history of previous GDM, macrosomia and congenital anomalies, BMI ≥ 25, pregnancy-induced hypertension, family history of diabetes, history of stillbirth, PCOS, history of abortion, age ≥ 25 years, multi-parity, and history of preterm delivery (18). Gestational diabetes is thought to be caused by the inability of the pancreas to produce insulin to handle excess sugar load during pregnancy. Women with GDM have a 50% chance of developing diabetes. Gestational diabetes increases the risk of complications for both mother and child during pregnancy, childbirth, and beyond. Furthermore, the disruption of maternal metabolism during pregnancy and periconception increases the risk of future diseases in children. An aberrant intrauterine environment caused by elevated maternal glucose levels is related to elevated risks for increased birth weights and metabolic disorders in later life, such as obesity or type 2 diabetes (19).

### 2.3 Preterm birth

Approximately 10% of all newborns are preterm. There are serious health risks associated with premature birth, including lifelong disabilities and high healthcare costs for children. Prevention of premature births is therefore a high priority, but the etiology of the majority of cases is not known. Apart from omics data, many other macro-level strategies have been suggested, including studying the effects of migration and of populations in transition, public health programs, tobacco control, routine measurement of length of the cervix in mid-pregnancy by ultrasound imaging, prevention of non-medically indicated late preterm birth, and identification of pregnant women for whom treatment of vaginal infection may be of benefit (20).

### 2.4 Mental health

Approximately 70% of perinatal women hide their mental health issues. Untreated mental health issues is a public health concern as it affects the physical and mental health of the entire family. Maternal mental health problems include antenatal and/or postnatal depression, anxiety, obsessive–compulsive disorder, postpartum psychosis, and post-traumatic stress disorder. Depending on the severity, different care or treatment plan is needed. Young mothers face mental health challenges during and after pregnancy including increased rates of depression compared to older mothers.

Prenatal depression risk factors include demographic measures (lower socioeconomic status, less education, non-marital status, unemployment, less social support, unintended pregnancy, partner violence, and history of child abuse) and physiological variables (cortisol, amylase, and pro-inflammatory cytokines and intrauterine artery resistance) (21). Income and marital status significantly moderated the relationship for depressive symptoms in late pregnancy, and stress in late pregnancy mediated the effects of marital status and satisfaction with relationship in early pregnancy. The effects of young maternal age, single marital status, low education, Aboriginal ethnicity, low income, poor relationship status, and high stress in early pregnancy were partially or completely mediated through smoking and drug use in late pregnancy in predicting depressive symptoms in late pregnancy in a longitudinal Canadian cohort study (22).

## 3 Machine learning and multi-omics

Advances in sequencing technologies have exploded in recent decades. Generation of data at various -omics levels (epigenome, transcriptome, proteome, and metabolome) is no longer a bottleneck in characterizing the disease states. The challenge is to integrate large multi-omics data in a meaningful way to understand functional wiring of the system under study. The term “omics” refers to the scientific domains involved in high-throughput measurements of biological molecules (DNA, RNA, protein, and metabolites). Associating omics-based molecular data with a clinical outcome of interest is the common objective of omics investigations. The justification is that by utilizing omics-based data, there is a possibility of creating predictive or prognostic models for certain medical conditions or diseases that are more accurate than what can be acquired through conventional clinical procedures. Machine learning, i.e., statistical approaches used to “learn” through training models using data and fitting models to data, is used to integrate and analyze the various omics data, enabling the discovery of new biomarkers. These biomarkers have the potential to help in accurate disease prediction, patient stratification, and delivery of precision medicine (23). Precision medicine, which predicts which treatment procedures are likely to be effective on a patient based on a variety of patient features and the context of the therapy, is the most common application of classical machine learning in healthcare (24). Deep learning or neural network models with numerous levels of features or variables that predict results are an example of the more advanced types of machine learning (25). Novel deep learning and machine learning approaches have been applied successfully recently to robustly predict patient survival subtypes using multi-omics data (26).

## 4 Multi-omics for diagnostics, prevention, and management

Maternal urine and blood are widely used for pregnancy monitoring due to the easy availability of these samples. Accordingly, many -omics technologies have been applied to these samples to find diagnostic markers but are yet to be used in clinical protocols. The plasma concentration of ADAM12 (A Disintegrin and Metalloproteinase-12) has been found to be altered in several pregnancy-related disorders, but the usefulness of ADAM-12 as a marker for adverse outcomes is still unclear (27). Glucocorticoid receptor co-chaperone gene sensitivity in peripheral blood gene expression diminished with the progression of pregnancy and increasing maternal depressive symptoms and may serve as a biomarker for risk of developing depressive symptoms during pregnancy (28). Lower allopregnanolone during pregnancy predicts postpartum depression, with every additional 1 ng/ml of second-term allopregnanolone resulting in a 63% reduction in the risk of developing PPD (29).

The umbilical cord blood (UCB) is in contact with all the fetal tissues and can reflect the state of the fetus (both physiological and pathological, if any), and UCB can be compared with maternal blood. UCB is an easily available biofluid of diagnostic value and poses small risks to donors. During fetal development, many regulatory substances are exchanged and released into UCB, which could reflect the physiological and pathological condition of the fetus and pregnancy status and therefore have a prognostic value (30). Changes in UCB proteins (alpha-fetoprotein, adiponectin, and leptin) are used as diagnostic and therapeutic parameters to monitor fetal and neonatal disorders.

Genomics: Genome-wide association studies (GWAS) are widely applied to identify causal genetic factors for both health and disease traits. For example, a GWAS study of 281,416 individuals without diabetes identified 242 loci associated with glycemic traits (31). There are a few leading studies on the genetics of PCOS (32, 33). The first GWAS included a group of 744 women with PCOS and 895 controls of Han Chinese women. The two replication cohorts included 2,840 women with PCOS and 5,012 controls, and 498 women with PCOS and 780 controls, respectively. The PCOS GWAS identified three main gene loci at chromosome 2p16.3, 2p21, and 9q33.3. Postpartum depression has been called a disease of modern civilization because of the mismatch between current and evolutionary historical perinatal circumstances (34). About half of the variability in perinatal depression can be explained by genetic factors, significantly more than the heritability of non-perinatal depression at 32% (35). Genomics analyses thus show promise to find biomarkers as an objective index to facilitate diagnosis (e.g., postpartum depression) removing subjectivity of the medical practitioner.

Development during pregnancy is significantly influenced by epigenetic processes. Methylomics revealed that the fetal methylation profile inferred from maternal plasma resembled that of the placental methylome. The diagnosis of fetal trisomy 21 is a possible clinical use for maternal plasma bisulfite sequencing (36). Epigenetic changes during the pregnancy monitored by DNA methylation variation at HP1BP3 and TTC9B without a previous psychiatric diagnosis could predict the risk of postpartum depression with an area under the curve (AUC) of 0.81 (37). A substantial number of methylation differences reside in non-coding regions of the genome that are associated with the overexpression of long non-coding RNAs (lncRNAs). These lncRNAs expressed abnormally in placenta from preeclamptic pregnancies and they may play a role in the functional development of preeclampsia (38).

Proteomics: Urinary proteomics can identify biomarkers for preeclampsia more than 10 weeks before clinical presentation. Two such markers are fragments of SERPINA1 and albumin, upregulated in women with preeclampsia but downregulated in gestational hypertension. Increased levels have also been seen in inflammatory conditions such as vasculitis and cardiovascular disease. This profile has a better predictor compared with the sFlt1:PIGF ratio and urine protein:creatinine ratio. Chen and colleagues also had similar findings in women with preeclampsia and gestational hypertension as compared with normal pregnancy *via* proteomics (32). Twenty-two highly replicable candidate biomarkers that were significantly different between women with GDM and controls across various proteomic platforms were found, which were most strongly linked to pathways related to complement and coagulation cascades (39). Models built from plasma proteomic data predict spontaneous preterm delivery with intact membranes with higher accuracy and earlier in pregnancy than transcriptomic models (40).

Metabolomics: Metabolomics is a rapidly growing technology that characterizes the complete collection of metabolites or small molecules found in an organism or in its cells, tissues, and biofluids. Odibo described four metabolites (hydroxyhexanoylcarnitine, alanine, phenylalanine, and glutamate) that increase during preeclampsia (41). The serum metabolomic profile of postpartum depression identified increased levels of glutathione-disulfide, adenylosuccinate, and ATP, which are associated with oxidative stress, nucleotide biosynthesis, and energy production pathways (42). Ten metabolites were differentially expressed in PPD urine in a small metabolomic study (43).

Other omics: Though previously mentioned omics approaches are most widely used, there are many other relevant ones. Metallomics analysis revealed the increase of several metals (As, Cd, Ni, Pb, Al, Mn, and Co) in the preeclampsia placenta. An Rb deficiency might be related to preeclampsia occurrence. The comparable concentrations of Ca, P, and Mg in controls and cases indicate a subordinate role of mineralization in preeclampsia, despite the disorder’s hypertensive origin (44). Transcriptomics variations related to preeclampsia in early pregnancy was detected in a study (45) that identified genes associated with preeclampsia overlap with transcriptome signatures associated with maternal asthma, vitamin D insufficiency, and excess BMI.

Microbiome: Both gut and vaginal microbiome are highly relevant to pregnancy. The study of the microbiota has been transformed by the sequencing-based metagenomics research on the human microbiome because it can produce an extensive library of microbial genomes across a variety of ecological niches within large hosts like humans (46). Microbiota studies during pregnancy are timely and highly relevant for many conditions. For example, increased risk of pelvic inflammatory disease is linked to an imbalance in the vaginal microbiome, and polymicrobial etiology makes diagnosis and treatment problematic (47). A microbiome study for preterm birth revealed that women who delivered preterm exhibited significantly lower vaginal levels of *Lactobacillus crispatus* and higher levels of BVAB1, *Sneathia amnii*, and TM7-H1, and preterm-birth-associated taxa were correlated with proinflammatory cytokines in vaginal fluid (48).

### 4.1 Biomarkers for maternal health and aging

Birth rates among female birth cohorts are declining, and childbearing occurs at an increasingly older age. An increasing proportion of women want to have children after 40 years of age, but more women fail to meet their fertility intentions expressed at 34–36 years of age (49). Sterility was unlikely the main cause for this as sterility was estimated at approximately 1% and it did not change with age (50). Typically, women have a biological reproductive span of approximately 37 years but fertility is not uniform through the reproductive span (51). By the age of 30, fertility begins to decline and the decline accelerates further to be exhausted by the age of 45 in most women. Moreover, aging increases risk to other diseases including of the reproductive system such as uterine fibroids and endometriosis. Almost all pregnancy complications are linked to age. Pregnancy at advanced maternal age has an increased risk of gestational diabetes or preeclampsia as the disease susceptibility rises with age. Furthermore, the likelihood of genetic and chromosomal abnormalities of fetus is highly increased with maternal age.

The genetic mechanisms causing pathway changes to affect age-related infertility are now being identified. One such process is DNA damage response (DDR) pathways, and experimental manipulation of DDR pathways highlighted by human genetics increases fertility and extends reproductive life in mice (52). Another process is the clonal hematopoiesis of indeterminate potential (CHIP), an age-related expansion of hematopoietic cells with leukemogenic mutations without detectable malignancy (53). The presence of CHIP is associated with a higher risk of atherosclerotic cardiovascular disease, cancer, and mortality. Accordingly, CHIP is strongly linked to age acceleration in multiple aging clocks (54). Early menopause is an independent risk factor for cardiovascular disease in women, but mechanisms underlying this association remain unclear. Early menopause, especially natural early menopause, was independently associated with CHIP among postmenopausal women (55). Across individual lifestyle factors, having a normal body mass index was strongly associated with a lower prevalence of CHIP but a healthy lifestyle score was not associated with CHIP (56). A cohort study of over 44,000 UK biobank participants showed that the prevalence of CHIP decreased as diet quality improved from unhealthy to intermediate to healthy (57). In summary, CHIP might provide a proxy for overall health status and could potentially be used to identify a population at high risk for adverse outcomes including high risk of all-cause mortality.

## 5 Machine learning and predictive models

Humans are multi-cellular complex organisms and the biological processes within single cells are complex. Most current omics technologies allow genome-wide readout only at a single level (genomic, epigenomic, transcriptomic, proteomic, metabolomic, etc.). Multi-omics refers to combining two or more omics datasets for data analysis, visualization, and interpretation to understand the biological processes behind disease states. With advances in multi-omics technologies, a wealth of large genomics data, including at the single-cell level, is becoming available, and more and more atlas-based initiatives help unravel fundamental cellular biology (**Table 1**). The combined use of heterogeneous and complementary multi-omics assays can reveal interactions between modalities that are key to biological processes. Only through the integration of multiple types of data across molecular, cellular, spatial, and population scales can biological systems be fully characterized. The rapid progress of digital and -omics technologies, together with the advances in machine learning, is now building up momentum for precision medicine to apply the personalized healthcare solutions developed under research setting into clinical practice. Glycemic traits are used to diagnose and monitor type 2 diabetes (T2D) and cardiometabolic health. A recent machine learning approach could predict the risk of cardiovascular disease in patients with T2D with approximately 80% accuracy using the administrative data. Furthermore, a 1-year incident hypertension risk model using electronic health records achieved an accuracy of approximately 90% (62).

**Table 1:** A representative list of studies on pregnancy complications using omics data.

Prediction trait	Variables	Sample size	Publication
GDM	Proteomics	1,779	(39)
Preeclampsia	Metallomics	40	(44)
GDM, DM Type II	Genetic data (GWAS)	5,485	(58)
Preeclampsia	Transcriptomics, physiological and clinical	157	(45)
Gestational age	Multi-omics	51	(59)
Preeclampsia	Epigenetic modifications	12	(38)
Postpartum metabolic syndrome	Biochemical data	847	(60)
Preeclampsia	Genomics	43	(61)

Machine learning techniques are of high value if integrated properly in maternal and perinatal care. There is a demographic trend towards more and more women requiring medically assisted birth partly due to increasing maternal age and maternal BMI. Though machine learning shows promise to build predictive models, there have been limited efforts in this area (**Table 2**). There is a huge potential in these predictive models to the healthcare system for the hospital management as well as for developing preventive care. A recent retrospective cohort study used machine learning on electronic medical record data from 303,678 deliveries to successfully predict approximately 52% of obstetrical complications (89). Low- and middle-income countries contribute to most fetal and neonatal deaths. Using machine learning on the cohort data for over 500,000 pregnancies in the low-resource setting, the predictive models achieved predictive accuracy for both intrapartum stillbirth and neonatal mortality with an AUC value of 0.71 and found that birth weight was the most important predictor for neonatal mortality (88). Preterm birth is the single most important contributor of neonatal and perinatal deaths, and the causal factors behind most cases remain unexplained. Machine learning from transcriptomics and proteomics profiling of plasma and metabolomic analysis of urine allowed accurate predictions of preterm birth, with an AUC of 0.83 (87). Despite decades of research into preeclampsia, clinicians have not been able to predict the medical condition prior to onset of symptoms. Some predictive markers are identified and tested such as prediction of early and late preeclampsia from maternal characteristics, uterine artery Doppler, and markers of vasculogenesis during the first trimester of pregnancy (90). Changes in maternal plasma soluble Flt-1, soluble endoglin, and placenta growth factor between the first and early second trimester combined with clinical characteristics also showed promise for predicting early-onset preeclampsia (91). Recently, a machine learning-based multiomics model for preeclampsia risk by analyzing six -omics datasets from a longitudinal cohort of pregnant women reached high accuracy with an AUC of 0.94 (92).

**Table 2:** Overview of predictive models of female reproductive health complications using machine learning approaches.

Prediction trait	Variables	Sample size	Publication
PCOS	Clinical, metabolic, and hormonal	>100	(63)
PCOS	Physical and psychological parameters	>500	(64)
PCOS	Body composition	<100	(65)
PCOS	Scleral images	>500	(66)
PCOS	Gene expression	>100	(67)
Deep endometriosis	Blood plasma markers	>100	(68)
Endometriosis	Serum miRNA	100	(69)
Deep endometriosis	Transvaginal sonography, MRI	>100	(70)
Preeclampsia	Physiological parameters	>500	(71)
Pregnancy hypertension	Physiological parameters	>1,000	(72)
Preeclampsia	Angiogenic biomarkers	>5,000	(73)
Preeclampsia	Angiogenic factors	>5,000	(74)
Preeclampsia	Maternal characteristics	>500	(75)
Preeclampsia	Maternal characteristics	>10,000	(76)
GDM	Electronic health records	>40,000	(77)
GDM	Clinical data and biomarkers	<1,000	(78)
GDM	Genetic data	>1,000	(79)
GDM	Clinical and biochemical biomarkers	>200	(80)
GDM	Clinical and biochemical data	>1,000	(81)
GDM	Maternal characteristics	>3,000	(82)
Stillbirth	Maternal demographic and clinical data	>100,000	(83)
Stillbirth	Birth registry data	>1 million	(84)
Stillbirth and preterm birth	Maternal demographic and clinical data	>10 million	(85)
Perinatal death	Birth cohort	>40,000	(86)
Preterm birth	Multi-omics data	<100	(87)
Perinatal mortality	Registry data	>500,000	(88)
Birth complications	Electronic health record	>300,000	(89)

Similar to PCOS, endometriosis, and pregnancy-related complications discussed above, we summarize below the utilization of large-scale data analysis and omics techniques towards diagnosis, prognosis, and management of other prevalent female reproductive disorders, such as premature ovarian insufficiency, uterine fibroids, and sexual dysfunction.

### 5.1 Premature ovarian insufficiency

Premature ovarian insufficiency (POI) is pathogenic depletion of follicles before the age of 40 (93). POI is associated with fragile X premutation and X chromosome genomic abnormalities. To rule out premutation carrier status, FMR1 gene testing for the CGG repeat in the gene’s 5’ untranslated region is currently the sole gene recommended for clinical testing in women with POI (94). Also, many genes implicated in early menopause such as the aspartate/glutamate solute carrier family 25 member 13 (SLC25A13), the mini-chromosome maintenance complex component 6 (MCM6), the corticotropin-releasing hormone receptor 1 (CRHR1), and the MB21D1 or C6ORF150 substantially correlated with age at early menopause (95). In order to assess the customized risk of POI following chemotherapy, Chung et al. constructed a machine learning-based model that had an accuracy of 88% (area under the ROC 0.87, 95% CI: 0.77–0.96; *p* < 0.001) (96). An enhanced mean shift algorithm based on artificial intelligence (AI) technology was used to process ultrasound images in women with idiopathic POI, where the functional condition and hemodynamics of patients’ ovaries were clearly visible on the transvaginal color Doppler ultrasonography (97). Another study demonstrated that building diagnostic methods for POI prediction may be accomplished using artificial neural networks, where the generalization ability of the train set, validation set, and test set was validated, with a prediction accuracy of over 90% in the test, train, and validation sets (98).

### 5.2 Uterine fibroids

Fibroids are non-cancer growths in or near the uterus, also called uterine myomas or leiomyomas (LM) (99). A pathogenomics study of the development of uterine fibroids led to the identification of several new gene networks and biological processes, as well as information about the inter-cell matrix’s effect on LM growth and the role of microRNAs in its control. The side population of female reproductive system embryonic myoblasts, which eventually gave rise to numerous tiny and medium fibroids, is thought to be caused by MED12 gene alterations, whereas HMGA2 gene hypomethylation and, thereby, overexpression, which is facilitated by hypoxia, muscular stress, or chromosomal instability/aberrations, were the primary causes of the solitary and often large-sized fibroids in LM SC (100). To distinguish between a normal and disordered uterus from the TCGA-UCEC dataset, an automated technique based on VGG 16 of deep learning classification models was used. The model’s accuracy in predicting the kind of uterine fibrosis from image data is 98.5% (101). Using neural networks, the Fibroid Disease Prediction System (FDPS) was created. The chance that a patient will develop fibroid disease was predicted using the FDPS method based on 10 medical characteristics for prediction, including age, excessive bleeding, marital status, being single or married, and pelvic discomfort. The neural network accurately predicted fibroid illness in approximately 98% of cases (102).

### 5.3 Female sexual dysfunctions

Female sexual dysfunction (FSD) is characterized as a disturbance of sexual desire, arousal, or orgasm, and/or sexual pain, which causes emotional suffering and has an effect on quality of life and interpersonal interactions (103). Despite recent efforts, e.g., the most current version of the Diagnostic and Statistical Manual of Mental Disorders, edition 5, abandoning the outdated linear model of diagnostic categories and collapsing previous classifications of female sexual disorders, FSD remains largely understudied (104). In the epigenome-wide analysis of DNA methylation in whole blood samples, two differentially methylated CpG sites (cg09580409 and cg14734994), corresponding to MGC45800 and the threonine synthase-like 2 gene (THNSL2), respectively, were detected for general sexual functioning. Additionally, putative physiologically relevant candidates for sexual pleasure (solute carrier family 6 member 19, SLC6A19) and desire (CUB and zona pellucida-like domains 1, CUZD1) were found. THNSL2 and SLC6A19, which have been connected to obesity and weight gain, may be new options for FSD (105).

## 6 Current opportunities, challenges, and future prospects

Gene, protein, and metabolite networks have heightened importance as individual molecules have a limited effect on disease analysis (1%–10%) compared with panels of multiple markers. In conjunction with other omics data types, such multiple marker panels could lead to new diagnostic and prognostic tests, and are relevant to assessing novel therapeutic approaches. Important knowledge gaps in practice to successfully implement multi-omics approaches include incompleteness of the molecular interactome, challenges in identifying key genes within genetic association regions, and limited applications to human diseases (106). Researchers have utilized a variety of AI-based techniques (machine and deep learning models) for diagnosing diseases, including the K nearest neighbor (kNN), support vector machine (SVM), decision tree, logistic regression, fuzzy logic, and artificial neural network (107). Deep learning for illness diagnoses needs more research, both to process vast amounts of medical data more quickly and to increase the likelihood of producing results that are satisfactory (108). A crucial part of precision medicine is measuring the stability of molecular profiles over time. A study of 100 healthy individuals analyzed blood-based molecular profiles, including proteomics, transcriptomics, lipidomics, and metabolomics, which found high variation between individuals across different molecular readouts, while the intra-individual baseline variation was low (109). In summary, there is support for the personalization of health, and comprehensive omics profiling in a longitudinal fashion is needed for precision medicine.

Not many AI or machine learning applications to improve women’s health are in clinical practice as yet, particularly during pregnancy (110). AI tools are promising for researchers and clinicians alike in terms of producing sound results and enhancing care at every stage of pregnancy (111). AI, machine learning, and data mining could be of immense help in personalized management, follow-up of pregnant women, and handling their clinical and epidemiological data, computational resources, scalability, privacy, and ethical concerns. These prospective technological advances in remote monitoring of pregnant women would provide conceptual and analytical framework to analyze the complex interplay between various biological modalities that govern preterm birth and other pregnancy-related pathologies. A recent literature review of the use of AI in medicine noted very little identifying and optimizing strategies for engagement, essential for AI to meaningfully benefit patients and other end users (112). In medicine, artificial neural networks (ANNs) (113) are widely used to learn and analyze imprecise pieces of information, and analyze nonlinear data and past examples for the assessment of pregnancy risk and prediction of APOs (114). Digital technology offers hope and allows pregnant women to stay away from hospitals and improves antenatal care for those who can get access; e-diagnostics will be promising for them during the COVID-19 pandemic. The development of direct-to-consumer platforms supports gestational disease management (115), remote health monitoring (116), low-resource prenatal care (117), text messaging, antenatal risk assessment (118), fetal health status prediction (119), preeclampsia prediction, and perinatal depression (120). Genetic testing helps identify a woman’s likelihood of passing on a genetic disorder. Traditionally, genetic testing is typically carried out under the supervision of a genetic healthcare professional in a clinical setting. Due to the genetic curiosity, a brand-new category of genetic testing services has emerged, recently known as “direct-to-consumer genetic testing” or DTC-GT. DTC-GT will allow expectant mothers to learn whether there is a chance that their children may have a genetic issue without the use of genetic health professionals or lengthy wait times for hospital consultations (121).

The COVID-19 pandemic profoundly affected the lives of the global population. It is known that stress and psychological distress can affect women’s menstrual cycles. Observational studies have demonstrated that many have experienced reproductive health disturbance as a result of the COVID-19 pandemic. During the epidemic, a large number of maternal mental health problems along with social issues such as domestic violence and loss of income have been documented. There was also a significant increase in suffering from mental health symptoms, as well as weight gain, longer working hours, and an unhealthier diet. On the other hand, a minority of women have described improvement in their reproductive health and lifestyle over the course of the pandemic. Thus, the COVID-19 pandemic provides a unique chance for epidemiological studies on maternal mental health, and social services for pregnant women and mothers.

## 7 Concluding remarks

**Figure 1:**
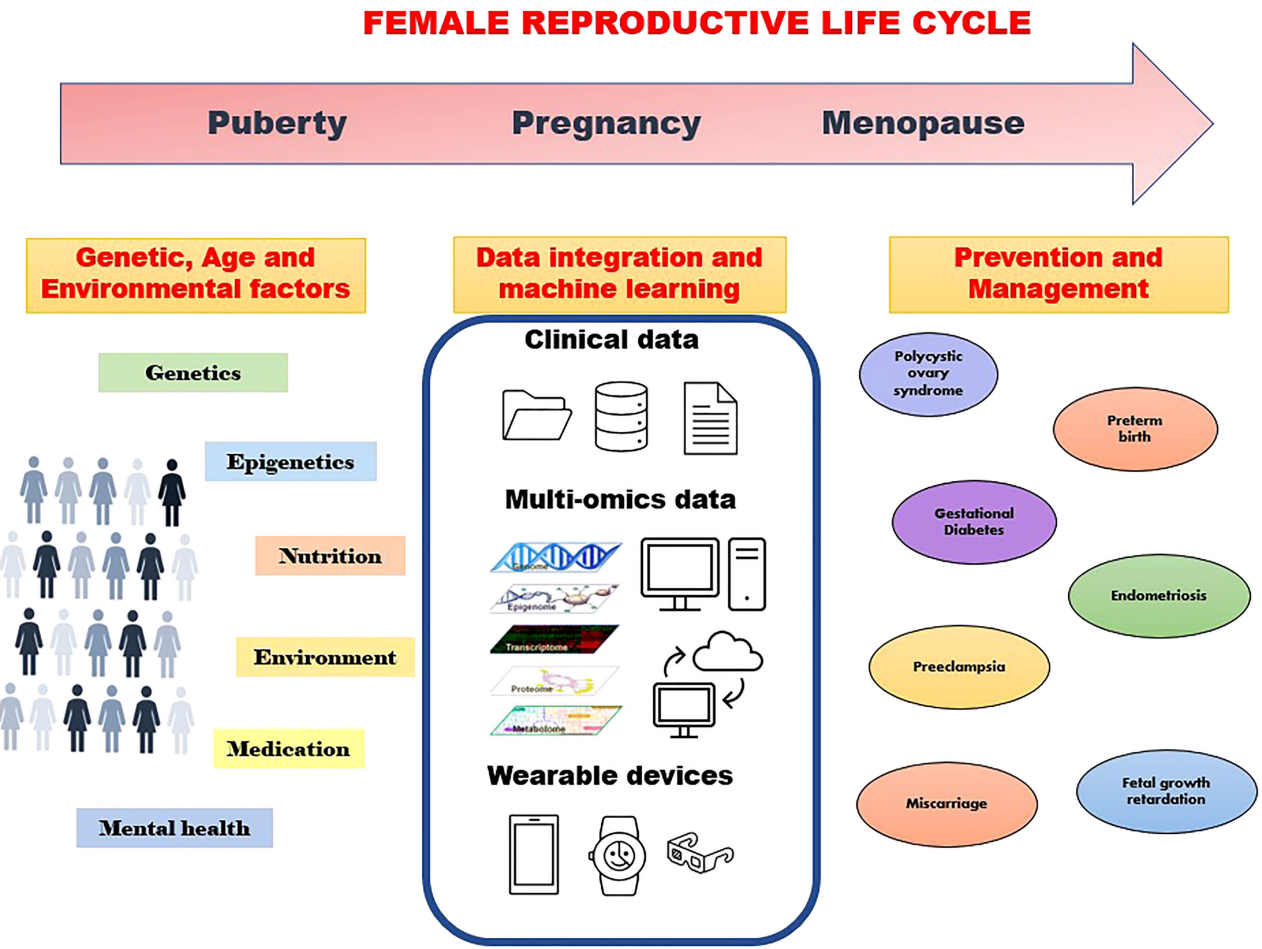
Machine learning approaches using various data to understand genetic and environmental factors towards prevention and management of disorders through the family reproductive life cycle.

Integration of epidemiology, genomics, epigenomics, transcriptomics, proteomics, metabolomics, metagenomics, and imaging data through bioinformatics will thus enable the identification of disease markers at different body organizational levels from cells, tissues, and organs to the individual level (**Figure 1**). Furthermore, in the era of smart digital mHealth technology during pregnancy, remote monitoring and early prediction of onset of many major pregnancy complications is feasible. WHO and Harvard have developed the Open Smart Register Platform allowing health workers to electronically register and track population health. It helps to deliver a powerful and dependable application to frontline health workers, empowering them to more effectively deliver and account for the care they provide to their clients (https://smartregister.org/). Many mobile applications are now available to track maternal health, with a global women’s health device market size of over US$25 billion in 2020. Telemonitoring of pregnancy using a digital health platform will be of help in enhancing antenatal care and will be a cost-saving approach to antenatal care. Telemedicine gained pace during the pandemic out of necessity, but the technology for affordable e-devices (e-Diagnostics) and point-of-care medical testing is still in infancy. Thus, despite the huge potential, we are still a long way to go from utilizing the full potential of omics and digital technologies in the health sector including women’s health.

## Author contributions

SK and AJ did the literature mining, analysis, synthesis, and writing of this review. All authors contributed to the article and approved the submitted version.
